# An unusual role for the phytyl chains in the photoprotection of the chlorophylls bound to Water-Soluble Chlorophyll-binding Proteins

**DOI:** 10.1038/s41598-017-07874-6

**Published:** 2017-08-08

**Authors:** Alessandro Agostini, Daniel M. Palm, Franz-Josef Schmitt, Marco Albertini, Marilena Di Valentin, Harald Paulsen, Donatella Carbonera

**Affiliations:** 1 Department of Chemical Sciences, University of Padova, via Marzolo 1, 35131 Padova, Italy; 2 Institute of Molecular Physiology, Johannes Gutenberg-University, Johannes-von-Müller-Weg 6, 55128 Mainz, Germany; 3 Institute of Chemistry, Technische Universität Berlin, Straße des 17, Juni 135, 10623 Berlin, Germany

## Abstract

Water-Soluble Chlorophyll Proteins (WSCPs) from *Brassicaceae* are non-photosynthetic proteins which tetramerize upon binding four chlorophyll (Chl) molecules. The bound Chls are highly photostable, despite the lack of bound carotenoids known, in Chl-containing photosynthetic proteins, to act as singlet oxygen and Chl triplet (^3^Chl) quenchers. Although the physiological function of WSCPs is still unclear, it is likely to be related to their biochemical stability and their resistance to photodegradation. To get insight into the origin of this photostability, the properties of the ^3^Chl generated in WSCPs upon illumination were investigated. We found that, unlike the excited singlet states, which are excitonic states, the triplet state is localized on a single Chl molecule. Moreover, the lifetime of the ^3^Chl generated in WSCPs is comparable to that observed in other Chl-containing systems and is reduced in presence of oxygen. In contrast to previous observations, we found that WSCP actually photosensitizes singlet oxygen with an efficiency comparable to that of Chl in organic solvent. We demonstrated that the observed resistance to photooxidation depends on the conformation of the phytyl moieties, which in WSCP are interposed between the rings of Chl dimers, hindering the access of singlet oxygen to the oxidizable sites of the pigments.

## Introduction

An unusual chlorophyll (Chl) binding protein in plants belonging to the *Brassicaceae* family was identified by Murata *et al*.^1, 2^. This protein, called Water-Soluble Chlorophyll Protein (WSCP), strikingly differs from most (bacterio)Chl-containing proteins in that it is water-soluble rather than being inserted into membranes. The only other two water-soluble (bacterio)Chl-binding proteins known so far are the Fenna-Matthews-Olson (FMO) complex^3^ and the Peridinin-Chlorophyll *a*–Protein (PCP) complex^4^, however, unlike WSCP, they are both involved in photosynthesis.

**Figure 1:**
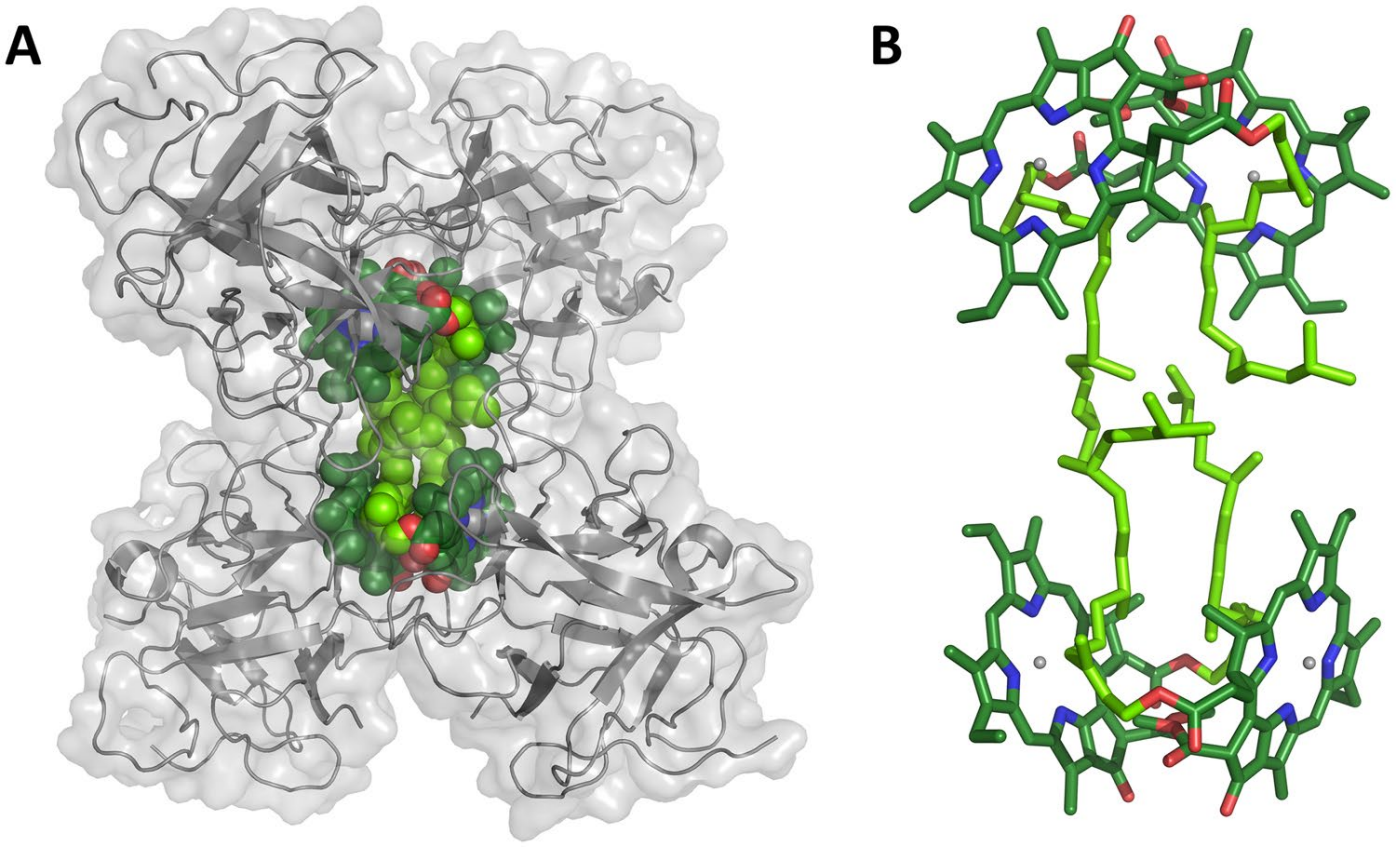
(**A**) Structure of *L. virginicum* tetrameric WSCP (PDB entry: 2DRE)^11^, with the polypeptide chains shown in gray cartoons, the protein surface in transparent gray, and the Chls *a* in spheres. The chlorin macrocycle is colored with carbon atoms in dark green, nitrogen atoms in blue, oxygen atoms in red and magnesium atoms in gray. The carbon atoms of the phytyl chains are colored in light green. (**B**) “Open sandwich” structure of Chl dimers shown in sticks.

Recombinant WSCP has been successfully reconstituted *in vitro* with Chls to a tetrameric complex that biochemically and spectroscopically is equivalent to native WSCP^5–10^. WSCP binds four molecules of Chl *a* (or *b*) per protein tetramer, as indicated by X-ray structures^11, 12^ (Fig. 1). The induction of WSCP synthesis under stress conditions^13–16^ and its ability to protect the bound Chls against photodynamic damage^5^, led to the hypothesis of its functional role as a Chl scavenger^10, 17^. Alternatively, Boeux-Fontvieille *et al*.^18, 19^ proposed that WSCP from *Arabidopsis thaliana* plays a role in herbivore resistance activation during greening and in programmed cell death at least during flower development. WSCP has been shown to have protease inhibitor activity^20, 21^, inhibiting for instance RD21 (RESPONSIVE TO DESICCATION-21)^19^, a papain-like protease^22, 23^.

The homotetrameric structure, showing a strict 222 symmetry in the X-Ray structure of *Lepidium virginicum* WSCP^11^, forms a hydrophobic cavity in the center, occupied by four Chls. Unusually^24–26^, these are bound from their *β-*side, a feature that could play a role in the stabilization of the tetramer^27^ as well as in the capability of the apo-protein to extract Chls directly from the thylakoid proteins^7^ where Chls are bound mostly from their *α*-side. The Chls share the same symmetric relationship of the homotetramer, with two Chls at a close distance (10 Å center to center) constituting an excitonic dimer^28^, weakly coupled to the other dimer (ca. 20 Å center to center distance) (Fig. 1). In the “open sandwich” configuration adopted by the Chls in the protein^9^, the excitation of the upper energy exciton band has a transition dipole moment that exceeds that of the lower energy band by almost one order of magnitude^28^. This feature strikingly contrasts the properties of the excitons in strongly coupled pigments of light harvesting complexes and RCs, having most of the oscillator strength in the lower energy exciton state^29^. The lower dipole strength of the lower energy exciton band leads to an increase of the lifetime of the excited singlet state with strong temperature dependency^30^, with a concomitant increase of the ISC probability and triplet yield^31^. In aerobic media, Chls are known to transfer the photoexcited triplet state energy to oxygen in less than 1 μs^32^, resulting in the formation of highly reactive singlet oxygen (^1^O_2_). The quantum yield of such an energy transfer in solution approaches 100%^33^, because the natural lifetime of the Chl triplet state (^3^Chl) is about 1.5 ms in most solvents^34^. In photosynthetic Chl-binding proteins, the formation of ^1^O_2_ is generally prevented by carotenoids located close to the Chls. These carotenoids quench ^3^Chl through triplet-triplet energy transfer^35–37^. In addition, carotenoids are also able to deactivate ^1^O_2_ directly^38, 39^. Although WSCP does not contain any carotenoid, the bound Chls have been shown to be effectively resistant to photobleaching^5^.

So far, the unexpected photostability of WSCP-bound Chls has not been satisfactorily explained. A shielding of the Chls from oxygen by the protein scaffold forming an efficient diffusion barrier has been proposed by Schmidt *et al*.^5^, however no clear evidence for this has been provided. In principle, the presence of strongly excitonically coupled dimers could lead to triplet states unable to photosensitize ^1^O_2_, as observed in some photosynthetic systems. This mechanism has been suggested to occur in chlorosomes^40^, where bacteriochlorophylls form aggregated states resulting in strong π-π interactions allowing the formation of triplet excitons sufficient to lower the triplet excited state energy below that of ^1^O_2_. It is known that also the solvent or the local pigment environment may shift the triplet state energies of several chlorin-type molecules by up to 0.11 eV^41^, possibly below the energy of ^1^O_2_. This latter effect has been proposed to underlie the photostability of the FMO complex^42^.

In order to get insight into the mechanism responsible for the photostability of WSCP, we performed a systematic study using electron paramagnetic resonance (EPR) techniques to determine the properties of the photoexcited ^3^Chl *a*, flash photolysis to measure the ^3^Chl *a* lifetime, and trapping experiments to evaluate the ^1^O_2_ yield.

## Results

### TR- EPR and pulse ENDOR

**Figure 2:**
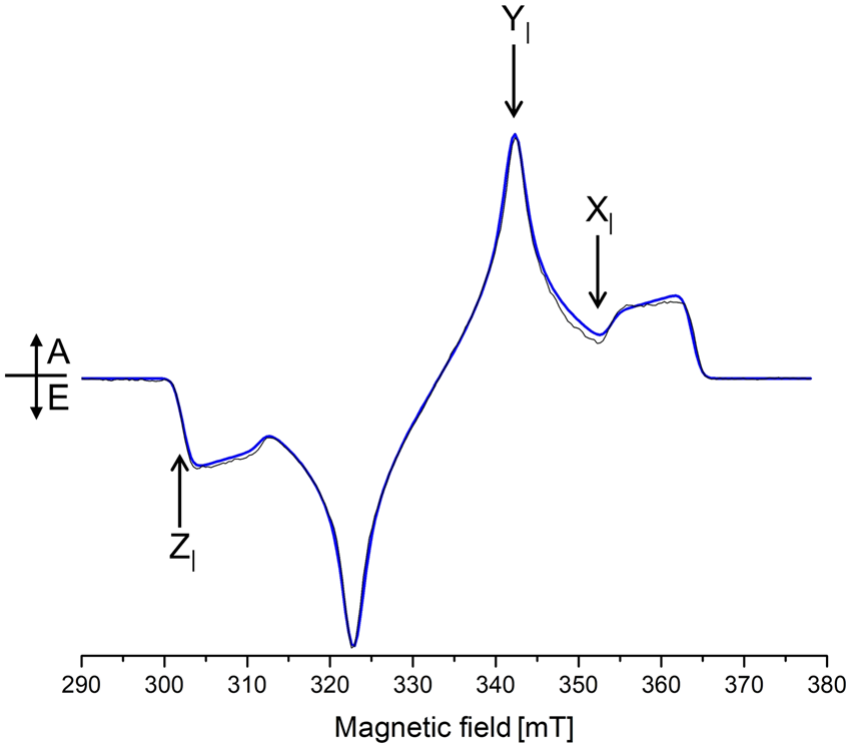
X-band TR-EPR spectrum of Chl *a* WSCP (black line), taken 150 ns after the laser pulse, at 130 K. Calculated spectrum (blue line) according to the parameters reported in Table 1. X, Y and Z represent the ZFS canonical orientations, only the transitions connecting the m_s_ = 0 to m_s_ = +1 levels are highlighted.

Illumination of recombinant *L. virginicum* WSCP reconstituted with Chl *a* leads to formation of ^3^Chl, which can easily be detected by time resolved-EPR (TR-EPR) at low temperature (Fig. 2). The polarization pattern of the spectrum is EAEAEA (A = absorption, E = emission), confirming the ISC population mechanism of the ^3^Chl. The population probabilities are more similar to those reported for ^3^Chl *a* in 2-Methyltetrahydrofuran (MTHF)^43^, rather than for ^3^Chl *a* in light-harvesting chlorophyll-binding proteins^44–48^.

Simulation of the spectrum (Fig. 2) has been obtained with the values reported in Table 1. The zero-field splitting (ZFS) parameters used in the simulation to reproduce the TR-EPR spectrum were similar to those reported for ^3^Chl *a* in MTHF and ^3^P680 in photosystem II particles^49^. The scheme of the Zeeman splitting of the triplet sublevels typical of chlorins, at the three canonical orientations of the triplet state (i.e. magnetic field parallel to the ZFS tensor axes X, Y, or Z)^50^, is reported in Fig. S1

**Table 1:** ZFS parameters and zero-field population probabilities (p_x, y, z_) of the ^3^Chl used in simulations of the TR-EPR spectrum. Other parameters: linewidth = 2 mT, g_iso_ = 2.0023.

	|D|		|E|		(p_x_,p_y_,p_z_)
	[mT]	[cm^−1^]	[mT]	[cm^−1^]	
WSCP Chl *a*	30.7	0.0287	3.8	0.0036	(0.12, 0.36, 0.52)
MTHF Chl *a* ^49^	30.5	0.0285	4.5	0.0042	(0.33, 0.56, 0.11)

The similarity of the D value of ^3^Chl *a* in WSCP with that measured in MTHF^49^, suggests a localization of the triplet state on only one of the two Chls constituting the Chl dimers bound to the protein, as observed for other Chl dimers in photosynthetic reaction centers of plants^49, 51^. However, a triplet state delocalization among Chls could still occur even in the absence of significant changes in the ZFS parameters with respect to those of the corresponding monomer, as recently observed in synthetic conjugated multimeric porphyrin systems^52^. On the contrary, electron-nucleus hyperfine coupling (hfc) constants, being directly related to the spin density distribution, strictly depend on the triplet delocalization. Indeed, transient Electron Nuclear Double Resonance (ENDOR) and pulsed ENDOR experiments performed on ^3^P680 clearly was crucial to demonstrating that the primary donor triplet in photosystem II is localized on a monomeric Chl species^49, 51^. Thus, we measured the proton hyperfine couplings on ^3^Chl *a* by means of pulse ENDOR spectroscopy.

**Figure 3:**
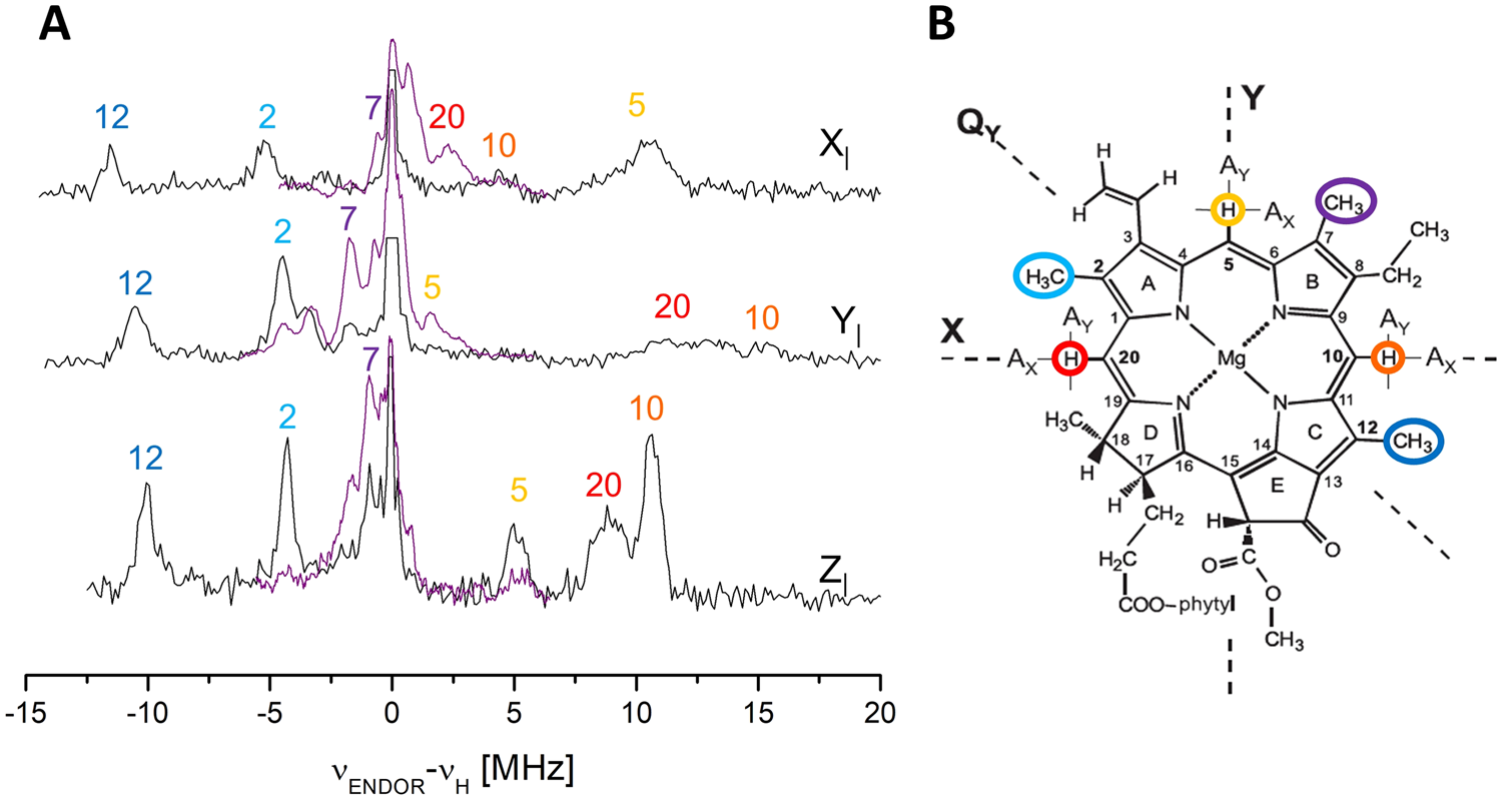
(**A**) X-band Pulse ENDOR spectra of WSCP ^3^Chl *a* for the X_I_, Y_I_ and Z_I_ EPR field positions (363.0 mT, 356.1 mT and 315.3 mT, respectively). Davies (black) and Mims (purple) spectra. Temperature 20 K. The frequency scale gives the deviation from ν_H_. The intense central ν_H_ lines in the Davies spectra have been cropped for better comparison. The assignments are labeled according to numbering and color code reported in panel *B*. (**B**) Scheme of Chl *a* structure with the orientation of the ZFS axes (**Z** not shown, perpendicular to the molecular plane). The protons corresponding to the ENDOR peaks in panel *A* are highlighted by circles.

Davies ENDOR spectra in WSCP were collected at field positions corresponding to orientations of ZFS axes of ^3^Chl parallel to the static field B_0_, yielding single crystal-like ENDOR spectra, from which the projections of the hfc tensors on the ZFS axis system can be extracted. For transitions connecting the m_s_ = 0 and m_s_ =  + 1 levels, positive hfcs give rise to ENDOR signals at frequencies lower than proton Larmor frequency (ν_H_), while negative hfcs are visible at higher frequencies with respect to ν_H_ (see black lines in Fig. 3A).

It is known that Davies pulse sequence does not allow to resolve signals with small hfcs. On the other hand, Mims ENDOR is particularly suitable for measuring small hfcs, but, since the spectra are intrinsically affected by blind spots, averaging at different τ values is required, making the experiment time consuming. Therefore, we performed Mims experiments only in a narrow frequency interval around ν_H_ (purple line in Fig. 3A) sufficient to obtain a complete set of the smaller hfcs.

The spectra were highly structured and well resolved, showing sharper peaks compared to the pulse ENDOR spectra of ^3^Chl *a* in MTHF reported before^49^. This is likely due to the lower inhomogeneous broadening experienced in the protein medium compared to the frozen glassy organic solution (MTHF). A similar effect was previously observed for the ENDOR spectrum of the primary donor ^3^P680^49^.

In the negative region of the spectra two main narrow peaks are distinguishable at each orientation selected by the field position, which can be attributed to protons belonging to rotating methyl groups, on the basis of the sign of the hfcs and on their small anisotropy. In particular, the highest hfc (a_iso_ =  + 10.7 MHz) is almost coincident with the value observed in the ENDOR spectrum of ^3^P680 (a_iso_ =  + 10.6 MHz), a monomeric ^3^Chl *a* ^49^, and assigned to methyl 12 (nomenclature as reported in Fig. 3B).

The other intense peak, with lower hfc values (a_iso_ + 4.7 MHz), may be assigned to methyl 2 on the basis of the hfcs reported for the Chl *a* cation^53^ and anion^54^ at this proton position, taking into account that, in the Hückel approximation, the hfc for a nucleus of a triplet state is expected to be the average of those observed in the respective cation (singly occupied HOMO) and anion (singly occupied LUMO) radicals, since in the triplet state the HOMO and LUMO are both singly occupied^49^. It is worth noting that this is the first time that the transition belonging to methyl 2 protons is clearly detected in a ^3^Chl *a* ENDOR spectrum. Methyl at position 7 is expected to give smaller hfcs, on the basis of the corresponding values measured for the cation and the anion radicals, which likely correspond to the less intense transitions visible in the Mims spectra (a_iso_ =  + 1.1 MHz), as reported in Table 2.

**Table 2:** hfcs of ^3^Chl *a* protons from the Davies and Mims ENDOR spectra shown in Fig. 3A.

	hfc component^a^ [MHz]	Assignment^b^					
		12^c^ (CH_3_)	2^c^ (CH_3_)	7^c^ (CH_3_)	5 (CH)	10 (CH)	20 (CH)
WSCP	A_X_	+11.6	+5.3	+0.6	−10.5	−4.4	−2.3
	A_Y_	+10.4	+4.5	+1.7	−1.6	−15.6	−12
	A_Z_	+10.1	+4.3	+0.9	−5.0	−10.7	−8.8
	a_iso_	+10.7	+4.7	+1.1	−5.7	−10.2	−7.7
MTHF^b^	A_Z_	+7.4	n.d.	n.d.	(−6.2)	−11.4	−7.2
^3^P680^b^	A_X_	+11.4	n.d.	n.d.	−7.1	−4.7	−3.3
	A_Y_	+10.0	n.d.	n.d.	−1.8	−14.8	−11.8
	A_Z_	+10.3	n.d.	n.d.	−5.5	−10.4	−8.4
	a_iso_	+10.6	n.d.	n.d.	−4.8	−10.0	−7.8
Chl *a* ^+•d^	a_iso_	+7.1	+3.0	+3.0	≤|0.6|	≤|0.6|	≤|0.6|
Chl *a* ^-•e^	a_iso_	+10.6	++5.3	−1.5	−4.7	−11.7	−4.4

^a^Principal components of the hfc tensor, axes collinear with the ZFS axes (X, Y, Z) within 20°.

^b^Based on Lendzian *et al*.^49^.

^c^Magnetically equivalent protons of the methyl group.

^d^Based on Huber *et al*.^53^.

^e^Based on Hoff *et al*.^54^.

In the positive part of the spectra three signals are present which can be assigned to three α protons, due to their sign and large anisotropy. Comparison with the previously published couplings relative to ^3^P680 and ^3^Chl *a* in frozen organic solvent, allows us to assign the observed transitions to the specific protons in the structure (Table 2)^49^. The small hfcs, which are well resolved in our Mims spectra, were neither assigned nor detected in previous pulse ENDOR studies, and will require a further computational analysis to reach a reliable and complete assignment.

The ^3^Chl *a* Z-axis is perpendicular to the molecular plane, and is expected to be also a principal axis of the observed hfc tensors for the -CH_3_ and α protons. Hence, changes in the spin density distribution due to delocalization of the triplet state between different Chls, should be directly reflected in the A_Z_ hfc values of the protons. Therefore, the absence of any reduction of the hfcs compared to ^3^P680 and ^3^Chl *a in vitro* clearly indicate that in WSCP the triplet state is localized on a single Chl molecule without any significant delocalization on the other Chls bound to the same protein unit.

### Flash photolysis

The characterization of the triplet state with EPR techniques ruled out the possibility of a photoprotective mechanism based on delocalization of the triplet state. Another important property of the triplet state that could be related to the slow photobleaching is the triplet lifetime. Since the intensity of the EPR signal depends mainly on spin relaxation processes and the ^3^Chl lifetime is usually not directly accessible by TR-EPR measurements^55^, we measured the ^3^Chl *a* lifetime by means of flash photolysis.

**Figure 4:**
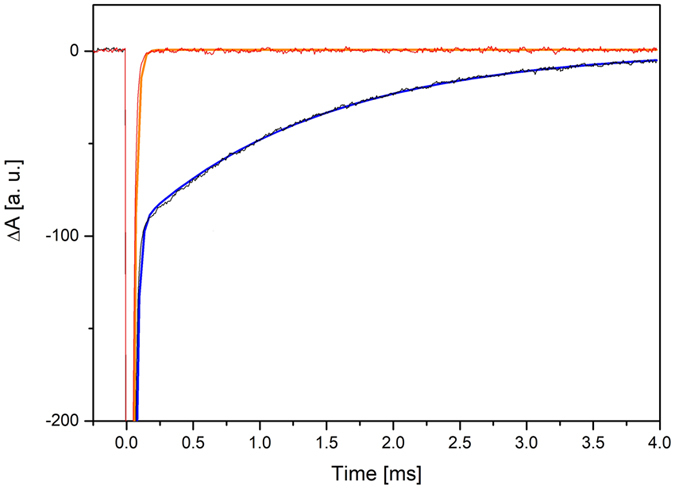
Recovery of the ground state absorption bleaching upon saturating excitation of WSCP Chl *a* in oxic (red) and anoxic (black) conditions, with the respective fitting curves. Saturating µs flash centered at the Q_y_. Low intensity probing light beam centered at the Soret band.

From the inspection of the flash photolysis spectra reported in Fig. 4, it is clear that the Chl ground state is depleted in the ms time range under anoxic conditions at least for a large part of all molecules, while, in oxic conditions, this time shortens to less than 10 µs (the limit of the time response of the setup). After subtraction of the lamp profile from the anoxic time trace, a lifetime of 1.35 ms and 40% amplitude for recovery of the ground state and then for the ^3^Chl *a* in WSCP was obtained, a value which is typical for ^3^Chl *a* in both organic solvent and proteins^34^. In addition, a further fast decay component near the resolution limit (10–20 µs) with 60% amplitude is found that results from spectral variations of the lamp profile which make it difficult to achieve a complete subtraction from the measured data and represent ground state depletion not correlated with inter system crossing and triplet formation. Hence an intrinsic shortening of the lifetime of the triplet state is not observed and cannot be invoked for explaining the photostability of WSCP. On the other hand the lifetime resulted to be very sensitive to the presence of O_2_, ruling out the hypothesis that the protein scaffold is shielding ^3^Chl *a* from interaction with oxygen, in agreement with our recent findings on *Brassica oleracea* WSCP^27^.

Schmidt *et al*.^5^ previously reported that light-induced ^1^O_2_ formation in WSCP was significantly reduced compared to that of free Chls in solution. This would imply that the shortening of the lifetime of ^3^Chl that we observed in the presence of O_2_ should take place without generating the oxidative species ^1^O_2_. In order to verify this possibility, we measured the photo-induced ^1^O_2_ yield in our system.

### ^1^O_2_ and photobleaching quantification

The photoproduction of ^1^O_2_ was measured using the fluorescent probe Singlet Oxygen Sensor Green® (SOSG). This sensor selectively reacts with ^1^O_2_, leading to an endo-peroxidized product with a highly increased fluorescence quantum yield. The measurements of ^1^O_2_ were accompanied by photobleaching measurements, performed on the same samples, by means of Chl fluorescence detection.

**Figure 5:**
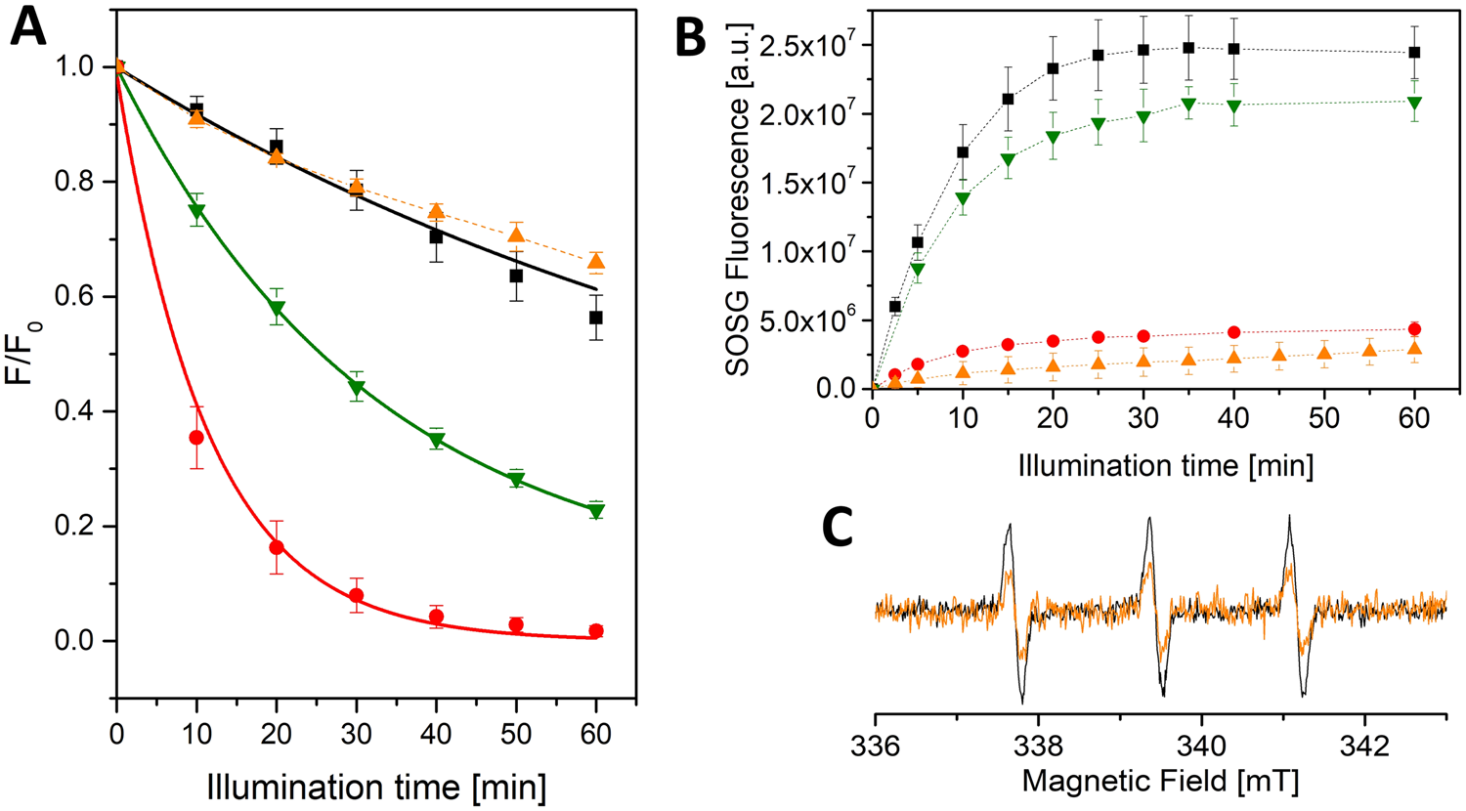
(**A**) Photostability of Chl *a* (black squares) or Chlide *a* (green inverted triangles) reconstituted WSCP and of LHCII (orange triangles) in comparison to Chl *a* in 2% (w/v) OG (red circles). The samples were illuminated with 500 µmol of photons m^−2^ s^−1^ for 0–60 min. before fluorescence measurements (excitation at 410 nm, emission F detected at the maximum of the emission spectrum). Emission before illumination: F_0_. Data are expressed as means (*n = *3). Mono-exponential fits are shown as continuous lines (fitting parameters in Table S1), LHCII data were not satisfactorily fit and the connecting line is reported in dash. (**B**) Singlet oxygen production of Chl *a* (black squares) or Chlide *a* (green inverted triangles) reconstituted WSCP and LHCII (orange triangles) in comparison to Chl *a* in 2% (w/v) OG (red circles). The samples were illuminated with 500 µmol red (λ > 590 nm) photons m^−2^ s^−1^ for 0–60 min before SOSG emission detection (excitation 480 nm, emission 530 nm). Data are expressed as means (*n = *3), with the connecting lines in dash. (**C**) Spin-trap EPR measurements of ^1^O_2_ production in Chl *a* reconstituted WSCP complexes (black) and LHCII (orange). TEMPO-formation detected after illumination at 150 μmol of photons m^−2^ s^−1^ for 60 min. Background signals were subtracted.

A remarkable photostability of WSCP was observed, as highlighted by the comparison with the major light harvesting complex (LHCII) of the photosynthetic apparatus in plants (Fig. 5A). The two Chl-binding complexes display comparable photobleaching, largely reduced in comparison to a reference sample containing free Chl *a* in octyl glucoside (OG) micelles. Thus, WSCP and LHCII exhibit a similar light tolerance although WSCP does not contain any carotenoids whereas LHCII binds four of them^56^.

Surprisingly and in marked contrast to the previous work of Schmidt *et al*.^5^ mentioned above, we detected an intense ^1^O_2_ production of WSCP (Fig. 5B), that reached a plateau only due to the consumption of the SOSG as highlighted by a control experiment in which upon addition of fresh SOSG, the fluorescence signal started again to grow upon illumination (data not shown). In comparison, LHCII exhibited a far smaller ^1^O_2_ production. A reference sample with unbound Chl *a* in OG solution displayed, under the same experimental conditions, an unexpectedly low ^1^O_2_ evolution. However, the reason for this behavior can be attributed to the concomitant high degree of photobleaching: due to the fast reaction with ^1^O_2_, the number of excitable Chls quickly decreases, and consequently less and less ^1^O_2_ is photosensitized at increasing time intervals.

Since these results, showing a high ^1^O_2_ yield in WSCP, were in contrast with those previously published, we performed also control spin-trapping experiments by Continuous-Wave (CW) EPR spectroscopy. When working in the same experimental conditions described by Schmidt *et al*.^5^, that is in the presence of *n*-dodecyl-β-maltoside (DM) (Fig. S2A), we also measured a low ^1^O_2_ yield. The different quantification of ^1^O_2_ measured by SOSG and spin probe methods may be related to the different repartition coefficients of the probes between the micellar and the aqueous phases. SOSG, which is a hydrophilic molecule, efficiently reacts with the ^1^O_2_ photosensitized by WSCP, whereas TEMP (2,2,6,6-Tetra-methyl-piperidine) is expected to be more concentrated in the micellar phase, in analogy with TEMPO (2,2,6,6-Tetra-methyl-piperidin-1-oxyl)^57^. Accordingly, we obtained a strong increase of the EPR signal of the spin trap upon removal of the DM from the WSCP solution (Fig. S2A), confirming that TEMP is sequestered away from the aqueous phase in the presence of the micelles. Therefore the underestimate of the ^1^O_2_ photosensitization in the previous report^5^ is due to the interfering presence of DM micelles.

Control experiments with free Chl *a* dissolved in an organic phase (ethanol) rather than in a micellar phase showed a 20% lowering (as estimated by double integration of the EPR signal) of the signal of the ^1^O_2_ spin trap (Fig. S2B). The decrease of the signal under this condition can be attributed to the fact that in homogeneous solution the increase in the local concentration, generated in micelles by the confinement of Chls and TEMP, is lost, and the probability of encounter between TEMP and ^1^O_2_ is consequently diminished.

In conclusion, our experimental approach based on two different methods for determining the ^1^O_2_ production led to the conclusion that ^1^O_2_ production in WSCP is not suppressed but is of the same order as that of free Chl and larger than that observed in LHCII (Fig. 5B and C), although a quantitative comparison between the two proteins is difficult, due to the fact that LHCII is a membrane protein while WSCP is water-soluble.

Prompted by the results on the triplet state characterization and singlet oxygen formation, we looked at the protein environment of the Chls, searching for a possible explanation of the scarce reactivity of the pigments towards ^1^O_2_. From the inspection of the crystallographic structure^11^, it appears that two tryptophans (Trp) are in close contact to the Chl macrocycle (namely W90 and W154). A possible role of Trp in photoprotection of photosynthetic proteins has already been hypothesized^58^, because of their high reactivity with ^1^O_2_^59, 60^. We mutated both of them to phenylalanine (Phe) residues, chosen because of the similarity of the side chains and the reduced reactivity with ^1^O_2_ reported for Phe^61^. The mutants were successfully reconstituted with Chl *a*, obtaining complexes with unchanged absorption and dichroism spectra in comparison to the wild type (see Fig. S3). Photobleaching of the mutants was very similar to that of the wild type, with a comparable loss of Chl *a* fluorescence (see Fig. S4) clearly indicating that neither of the Trp is involved in the photoprotection of the bound Chls, at least not by reaction with ^1^O_2_.

In order to study a possible photoprotective role of the phytyl chains of the bound Chls, which are interposed between the Chl planes, WSCP was reconstituted with chlorophyllide *a* (Chlide *a*), i.e. the phytyl-free analog of Chl *a*. The reconstituted pigment-protein complex was obtained in high yield and the purification via size exclusion chromatography showed that it was comparable in the size of the oligomers to the Chl *a* analog (see Fig. S5). Thus, although Chlide-containing WSCP is less stable than the Chl complex, the phytyls are not strictly required for the tetramerization of WSCP, in contrast to a previous report^5^.

It is well known that Chlide presents spectroscopic characteristics analogous to Chl, although some small differences have been reported^62^. The Chl *a* and Chlide *a* reconstituted complexes exhibited similar optical properties, as evident from the absorption and CD spectra (Fig. S6). Both Chl *a* and Chlide *a* upon binding to WSCP undergo a change in their absorption spectra compared to that of the pigments in organic solvents (see Fig. S7). The spectral similarity strongly suggests that the Chlides bound to WSCP are excitonically coupled in the same open sandwich configuration adopted by Chls. Interestingly, Chlide *a* WSCP showed a severe photobleaching (Fig. 5A), with an 80% loss of fluorescence after 1 h illumination. This is much higher than the photodegradation of Chl *a* WSCP under the same conditions (Fig. 5A), despite the similar spectral properties and although the ^1^O_2_ production was comparable between Chl *a*- and Chlide *a*-containing WSCP, as revealed by SOSG quantification (Fig. 5B).

## Discussion

In the present study, we used magnetic resonance techniques to characterize the ^3^Chl *a* populated in WSCPs under illumination. Our experimental results provide clear evidence for the triplet state to be localized on a single Chl molecule among the four interacting pigments belonging to one protein tetramer. This was demonstrated by the large values of the hfcs determined by ENDOR spectroscopy, which were very similar to those reported for monomeric ^3^Chl *a* in frozen organic solution and for the primary donor ^3^P680^49^. On the basis of these results we conclude that the photoprotective properties of the WSCP complex cannot be derived from a delocalization of the triplet state.

In marked contrast to previous findings^5^, we measured an intense photoproduction of ^1^O_2_ in WSCP complexes. The method used in the study of Schmidt *et al*.^5^, performed on *Brassica oleracea* WSCP, led to an underestimation of ^1^O_2_ due to the presence of detergent in the solution and to the limited solubility in water of the TEMP spin-trap. By modifying the experimental conditions we obtained spin-trapping results in agreement with SOSG quantification. Thus, the remarkable photostability of WSCP^5^, which is comparable to the one displayed by Chl-protein complexes in which the ^1^O_2_ production is efficiently avoided by the photoprotective role of carotenoids, has to rely on the capability of the system to resist to bleaching after ^1^O_2_ production. We have ruled out a possible involvement of two conserved tryptophans which are in van der Waals contact with the chlorin planes of the Chls.

**Figure 6:**
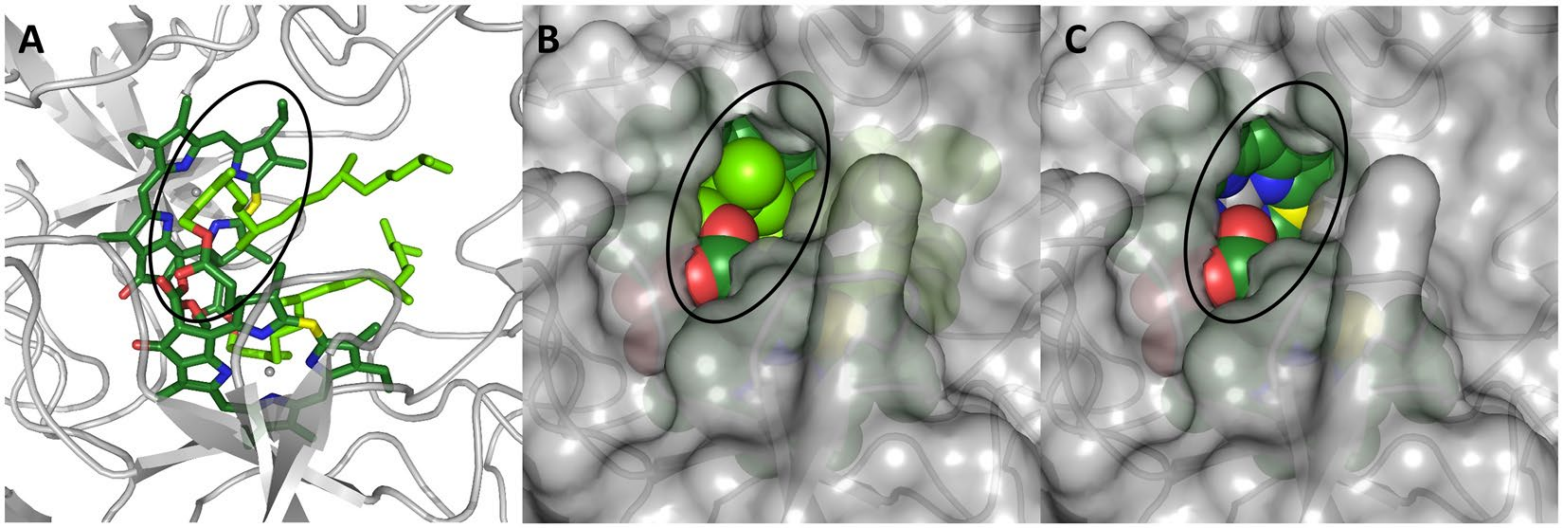
Close look of the structure of *L. virginicum* WSCP^11^, focused on the solvent-accessible part of the Chl molecule in the cavity (highlighted with a black ellipse). The same view is reported in the three panels, with the polypeptide chains in gray cartoons (protein surface in transparent gray in panels *B* and *C*) and the Chls *a* (*A* and *B*) or Chlides *a* (**C**) constituting a dimer shown either in sticks (**A**) or in spheres (**B** and **C**). Atoms colors as in Fig. 1.

The experiments performed on WSCP reconstituted with Chlide *a* clearly indicate that the presence of the Chl phytyls is required for the photostability of the system. A photoprotective action of the phytyls through a direct reaction with ^1^O_2_, i.e. acting as oxidizable species^63^, can be excluded, as this would have led to a photobleaching curve characterized by a change in slope, with a faster loss in fluorescence once all the phytyls are consumed by the reaction. Instead the profile of the Chl fluorescence keeps its mono-exponential decay unchanged also for long illumination times (see Fig. S8). The phytyls are intertwined between the Chl pairs constituting the open sandwich dimers (see Fig. 1). Interestingly this configuration brings them in van der Waals contact with the methine carbon atom of the Chl ring (number 20 in Fig. 3B, colored in yellow in Fig. 6) that is known to be the most susceptible site for reaction with ^1^O_2_ in the photobleaching of related Chls (Zn-Chl *a* ^64^ and BChl *c* ^65^).

The conformation of the phytyls seems to avoid an interaction of the methine 20 with the solvent (see Fig. 6B and C). The phytyls are also in close contact with the central Mg ions of the Chls and shield the Mg from the solvent. In a study conducted on bacteriochlorophylls (BChl) it has been proposed that interactions between oxygen and Mg increase the residence time of singlet oxygen near the BChls and, thus, lowers the photostability of the pigments^66^. Consequently, phytyls in WSCP may exert their photoprotective role also by masking the Mg ions rather than methine 20. It is likely that several structural components of WSCP contribute to the protection of the Chl moiety against access of singlet oxygen although clearly the phytyls play the most significant role. At the same time a small portion of the ring remains accessible (see Fig. 6B), which explains why the molecular oxygen can exchange triplet energy with ^3^Chl populating the ^1^O_2_.

It is worth noting that a photoprotective mechanism like this, with the reactive portions of the Chl macrocycle sterically shielded from the detrimental interaction with ^1^O_2_, has never been reported before in a protein system, but it is similar to that found by Bonchio *et al*.^67^ in a porphyrin-cyclodextrin supramolecular complex^68, 69^. These authors found that the encapsulation of the porphyrins inside a cyclodextrin matrix efficiently photoprotects the porphyrins from photobleaching, while preserving the capability of ^1^O_2_ sensitization. The available structural data of that system^68–70^ suggested that two cyclodextrins shield the methine carbon atoms avoiding their reaction with the photoproduced ^1^O_2_.

With regard to possible biological roles of WSCP, the protein has been proposed by Takahashi *et al*. as a Chl scavenger protecting cells from photodynamic damage by taking up unbound Chls and keeping them from producing ^1^O_2_^10, 17^. This hypothesis is not consistent with the present finding of WSCP as an active ^1^O_2_ producer. The remarkable photostability of the complex would render the Chl even more dangerous to the cell, since its elongated lifespan would lead to an even increased production of ^1^O_2_ over time.

The present observations suggest a different biological role of WSCP producing ^1^O_2_ as a biological signal for pathogen attack. Since in green tissues WSCP apoproteins are not located in chloroplasts but in ER bodies^10, 17, 71^, organelles involved in plant defense against pathogens and/or herbivores (for reviews see refs 72 and 73), WSCP complex formation can only occur upon cell disruption including organelle dismantling. WSCP apoproteins set free from the ER bodies would form tetrameric WSCP for instance by extracting Chls from thylakoid membranes^7^. In the presence of oxygen and light, these WSCPs would then produce ^1^O_2_.

It has been shown that the release of ^1^O_2_ triggers the activation of distinct stress response genes in *Arabidopsis* that are different from those activated by other reactive oxygen species like H_2_O_2_ or O_2_^∙−^^74, 75^. Hence ^1^O_2_ can function as a specific signal molecule inducing a specific set of plant stress responses^76^. The ^1^O_2_-triggered signaling pathways (for a review see ref. 77) are considered to result, depending on the level of produced ^1^O_2_, either in an acclimation response or in triggering programmed cell death programs (for a review see ref. 78). Moreover, certain hydroxy fatty acids due to non-enzymatic lipid oxidation by ^1^O_2_ were found to accumulate in *A. thaliana* leaves upon pathogen attack^79, 80^. Interestingly, application of hydroxy fatty acids as well as an induced production of ^1^O_2_ (by a photosensitizer) led to a strong accumulation of callose in leaves of *A. thaliana* ^81^, which has been shown to be a frequent response of plant cells towards pathogen attack^82^.

Consistently with the proposed role of WSCP as a producer of a ^1^O_2_ signal upon cell damage, the expression of the WSCP apoprotein is stimulated by pathogen attack^83, 84^ among other stress situations^13–16, 18, 21, 85, 86^. At extreme levels of ^1^O_2_, genetically controlled cell death responses are masked by genetically independent cell death reactions caused by the direct cytotoxicity of elevated levels of ^1^O_2_^87^ leading to the so-called accidental cell death^78^. In line with the latter notion, elevated levels of ^1^O_2_ produced by WSCP catalysis may even serve as an efficient weapon, directly damaging those pathogens that caused the formation of WSCP in the first place.

Another hypothesized biological role of WSCP is based on the observation that all *Brassicaceaen* WSCPs isolated so far possess a signature sequence of Künitz-type protease inhibitors (KTI)^6, 7, 10, 15, 17^. While some WSCPs have been reported to show inhibitory activity against proteases^19, 21, 88^, others did not^15, 89^. In etiolated apical hook tissue, Reinbothe and co-workers^18, 19, 90^ have recently identified a complex consisting of *A. thaliana* WSCP apoprotein interacting with RD21, a papain-like cystein protease involved in stress response and defense^22, 23, 91–93^. This interaction inhibits the cysteine protease activity of RD21^19^. Upon exposure to light, the RD21-WSCP complex dissociates, presumably due to pigment uptake of the WSCP apoprotein^18, 90^ suggesting a Chl/Chlide driven activation of the protease activity.

Clearly, WSCP functioning as a protease inhibitor de-activated by Chl binding would benefit from the photostability described in the present work. If the WSCP-bound Chls were rapidly photodegraded upon illumination, this would presumably unmask the protease inhibitor activity of the apoprotein again. It is worth noting that even at long illumination times, with a concomitant photodamage of some of the Chls bound within a tetramer, no WSCP complex degradation into monomers occurs (see Fig. S9). It should be noted that the two hypothesized WSCP activities in the plant defense, WSCP acting as a Chl-regulated protease inhibitor and WSCP acting as a producer of ^1^O_2_ as a signal for cell disruption, are fully compatible with one another and can be conceived as being performed in parallel.

## Conclusion

We have shown that Chls in WSCP efficiently produce ^1^O_2_ upon illumination but even so undergo little photooxidation. Our study solves the riddle of this photostability. Photoprotection requires the presence of the phytyl moieties of the WSCP-bound Chls, either by protecting those sites of the Chl macrocycle that are mostly susceptible to the ^1^O_2_ attack or by limiting the residence time of ^1^O_2_ close to them.

We propose that the photostability of the Chls bound to WSCP is required to allow playing the cell function, since Chls are needed to initially form the tetrameric structure of the protein, which in turn is instrumental for “sensing” and signaling the change in cellular conditions which leads to Chl release.

The quite simple mechanism of how the WSCP-bound Chls are photoprotected may inspire molecular design in the fields of photodynamic therapy or photocatalysis.

## Material and Methods

### Sample preparation

Chl *a* was isolated and purified from pea plants (*Pisum sativum*) according to Booth and Paulsen^94^. Chlide *a* was obtained using recombinant chlorophyllase from wheat (*Triticum aestivum*). Expression and purification of recombinant chlorophyllase was carried out following the protocol described earlier^95^. Chlorophyllase assay used for obtaining Chlide *a* is based on the method of Fang *et al*.^96^ with a few variations of the protocol. The reaction mixture containing 20 mM sodium phosphate (pH 7.8), 0.5 mg/ml chlorophyllase, 1 mM ascorbate, 5 mM MgCl_2_, 50% (v/v) acetone and 1.25 mg/ml Chl *a* was incubated for 4 h at 37 °C and 80 rpm. After adjusting to 70% acetone, the reaction mixture was centrifuged (10 min, 4 °C, 10,000 × *g*) and the supernatant subjected to Sep-Pak^®^ cartridges (Plus tC18, Waters) for purification via reversed phase chromatography. Chlide *a* was eluted by washing the cartridges with 4 ml of 70% acetone. After quantification via analytical HPLC, pigments were dried and stored under N_2_ atmosphere at −20 °C.

Pet24b plasmid with a gene encoding for a recombinant WSCP protein from *L. virginicum* possessing a C-terminal hexahistidyl tag^17^ was kindly provided by the group of Prof. Satoh (Toho University, Japan). The plasmid was transformed in *Escherichia coli* (BL21). Recombinant apo-protein expression and purification was carried out as described previously^27^ except the bacteria growth was carried out at 37 °C and kanamycin was used as the selection antibiotics. The purified apoprotein was reconstituted with a three-fold molar excess of either Chl *a* or Chlide *a*. Pigments were dissolved in Triton™ X-114 (TX-114) and added dropwise to the protein solution containing 20 mM sodium phosphate (pH 7.8) and 5 mM β-mercaptoethanol yielding a final concentration of TX-114 of 2% (v/v). After incubation for 30 min at 4 °C and 30 rpm (Intelli-Mixer, neoLab), phase separation was induced by increasing the temperature to 40 °C followed by a 5 min incubation step. The aqueous phase harboring the reconstituted WSCP complexes was collected after centrifugation (2 min, at room temperature, RT, 14,000 × *g*) and applied to a Superose™ 12 10/300 GL prepacked column (GE Healthcare) equilibrated with 20 mM sodium phosphate (pH 7.8) and operated by a BioRad NGC system at RT for further purification via size-exclusion chromatography (SEC). Peak fractions containing tetrameric WSCP were analyzed via absorption and circular dichroism (CD) spectroscopy. Recombinant LHCII was reconstituted and purified as described earlier^97, 98^. After sucrose density gradient purification, trimeric LHCII was collected and adjusted to 20 mM sodium phosphate (pH 7.8) + 0.2% (w/v) DM. LHCII complex integrity and functionality was verified by applying absorption, CD and fluorescence spectroscopy.

### TR-EPR and pulse ENDOR

Samples were concentrated to a Chl concentration of 700 μg/ml. Oxygen was removed by flushing nitrogen in the EPR sample tube before freezing. Residual oxygen was removed exploiting the glucose/glucose oxidase/catalase system^99^. Glycerol, previously degassed by several cycles of freezing and pumping, was added (60% v/v) just before freezing to obtain a transparent matrix.

X-band TR-EPR experiments were performed on a modified Bruker ER200D spectrometer with an extended detection bandwidth (6 MHz), allowing a response time of about 150 ns. Laser excitation at 532 nm (5 mJ per pulse and repetition rate of 50 Hz) was provided by the second harmonic of a Nd:YAG laser (Quantel Brilliant) in a high-Q cylindrical TE_011_ resonant cavity. The experiments were carried out with a microwave power in the cavity of 2 mW. The temperature was controlled with a nitrogen flow cryostat. The signal was recorded with a LeCroy LT344 digital oscilloscope, triggered by the laser pulse. The spectra were recorded averaging 400 transient signals at each field position. To eliminate the laser background signal, transients accumulated under off-resonance field conditions were subtracted from those on resonance. Simulations of the powder spin-polarized triplet TR-EPR spectra were performed using a program written in MatLab®, with the aid of the Easyspin routine (ver. 5.1.9)^100^, based on the full diagonalization of the triplet state spin Hamiltonian, including the Zeeman and magnetic dipole–dipole interactions, considering a powder distribution of molecular orientations with respect to the magnetic field direction^44^. Input parameters are the triplet state sublevel populations, the zero-field splitting (ZFS) parameters, the linewidth, and the isotropic g value.

X-Band Pulse ENDOR experiments were performed on a Bruker Elexsys E580 spectrometer. Laser excitation at 532 nm (10 mJ per pulse and repetition rate of 10 Hz) was provided by the second harmonic of a Nd:YAG laser (Quantel Brilliant) in a dielectric cavity. The temperature was controlled with a Helium cryostat (Oxford CF935) driven by a temperature controller (Oxford ITC503).

Davies-ENDOR experiments were performed using the microwave pulse sequence (flash-π-T-π/2-τ-π-τ-echo) with an inversion π pulse of 200 ns, T of 8 μs and a RF pulse of 6 μs. The detection sequence was similar to the field-swept ESE experiment but with τ of 300 ns.

Mims ENDOR experiments were recorded using the microwave pulse sequence (flash-π/2-τ-π/2-T-π/2-echo), with 16 ns pulse duration, in conjunction with an RF pulse of 6 μs duration, starting 0.8 μs after the second microwave pulse. The delay τ was variable, and the time T was 8 μs to accommodate the RF pulse. Mims ENDOR spectra were recorded at different τ values (from 160 to 240 ns) and added together to eliminate τ-dependent blind spots. Pulse ENDOR spectra were accumulated for ≈15 h.

### Flash photolysis

Flash-induced absorbance difference spectra on the µs to ms time domain were recorded at RT using a laboratory-built flash spectrometer as described by Gerken *et al*.^101^. The samples (2 ml; adjusted to an OD of 0.5 at 680 nm corresponding to around 7.5 µg Chls/mg) were placed in a 1 cm cuvette and excited with saturating flashes of ~20 µs in duration from a Xe flash lamp filtered by colored glass with transmission at 680 nm (model CS96–4 from Corning). Measuring light from a 55 W tungsten halogen lamp was passed through a monochromator with a spectral bandwidth of 3 nm, the optical cuvette and an interference bandpassfilter at 430 nm (AL 431) in front of a photomultiplier (EMI 9558BQ) coupled to a transient recorder (Tektronix TDS320) monitoring the bleaching of the Chl ground state absorption upon excitation. The time course of the absorbance changes in oxygen atmosphere was fitted to a biexponential decay including offset using Origin 8.0 ® software, after subtraction of the lamp profile. For measurements under anoxic conditions, oxygen was removed by using the glucose/glucose oxidase/catalase system^99^.

### Photobleaching

Photobleaching was measured by quantitating the loss in fluorescence intensity upon illumination with white light at 500 µmol of photons m^−2^ s^−1^. As a control, free Chl *a* was dissolved in a 20 mM sodium phosphate (pH 7.8) with 2% (w/v) OG. All samples were adjusted to a Chl *a* extinction of 0.005 (around 0.07 µg Chls/ml) at Qy maximum in either 20 mM sodium phosphate pH 7.8 (WSCP) or 20 mM sodium phosphate (pH 7.8) + 0.2% (w/v) DM (LHCII).

After excitation at 410 nm, Chl *a* emission (F) detected at the maximum of the emission spectrum (at 667, 674, 678 and 674 nm for Chl *a* WSCP, Chlide *a* WSCP, LHCII and Chl *a* in 2% (w/v) OG, respectively) was compared to the initial emission before illumination (F_0_). F/F_0_ was plotted against illumination time. The measurements were performed on a FluoroMax-2 instrument (Jobin Yvon-Spex) at RT using a cuvette with the dimensions of 5 × 5 mm and the following parameters: slits 1 nm, increment 1 nm, integration time 1 s.

### Singlet oxygen quantification

Quantification of ^1^O_2_ production was performed with SOSG (Molecular Probes, Eugene). SOSG is a fluorescent probe highly selective for ^1^O_2_ that increases its 530 nm emission band upon reaction with this species^102^. The different samples were diluted to the same integrated absorption area in the wavelength range 590–750 nm (around 2.5 μg Chls/ml) in the presence of 2 μM SOSG and 2% (v/v) methanol, and illuminated with red light (λ ≥ 590 nm) at 500 µmol of photons m^−2^ s^−1^ under stirring. Fluorescence yield of SOSG (λ_ex_ 480 nm, λ_em_ 530 nm, slits 0.5 nm, increment 1 nm, integration time 1 s, cuvette 1 × 1 cm) was determined after light treatment in order to quantify ^1^O_2_-dependent fluorescence increase.

Spin-trapping assays of ^1^O_2_ were also performed following the procedure described in Schmidt *et al*.^5^. All the samples were prepared in 20 mM sodium phosphate (pH 7.8) with a Chl concentration of 1 μg/mL, 100 mM TEMP, and 3.5% methanol (v/v). Samples were illuminated for one hour with white light at 150 µmol of photons m^−2^ s^−1^ on a water bath at 20 °C. CW EPR spectra of the samples were measured before and after exposure to light with a Bruker Elexsys E580 spectrometer. X-band spectra were recorded at 298 K with a modulation frequency of 100 kHz, a modulation amplitude of 0.2 mT, a microwave frequency 9.6 GHz, and a microwave power of 3.76 mW. Spin-trap concentrations were determined by performing double integration of the EPR signals.

## Electronic supplementary material

Supplementary Information
