# Supplementary material for: An unusual role for the phytyl chains in the photoprotection of the chlorophylls bound to Water-Soluble Chlorophyll-binding Proteins
Source: Sci Rep. 2017 Aug 8;7:7504. doi: 10.1038/s41598-017-07874-6 (PMC5548782; doi:10.1038/s41598-017-07874-6)
Supplement: Supplementary file 1 — Supplementary Information [file 41598_2017_7874_MOESM1_ESM.pdf]

Supporting Information

An unusual role for the phytyl chains in the photoprotection of the chlorophylls bound to Water-Soluble Chlorophyll-binding Proteins.

Alessandro Agostini[1,2‡], Daniel M. Palm[2‡], Franz-Josef Schmitt[3], Marco Albertini[1], Marilena Di Valentin[1], Harald Paulsen[2*] and Donatella Carbonera[1*]

[1]Department of Chemical Sciences, University of Padova, Via Marzolo 1, 35131 Padova, Italy,

[2]Institute of Molecular Physiology, Johannes-Gutenberg University Mainz, Johannes-von-Müller-Weg 6, 55128 Mainz, Germany,

[3]Institute of Chemistry, Technische Universität Berlin, Straße des 17. Juni 135, 10623 Berlin, Germany.

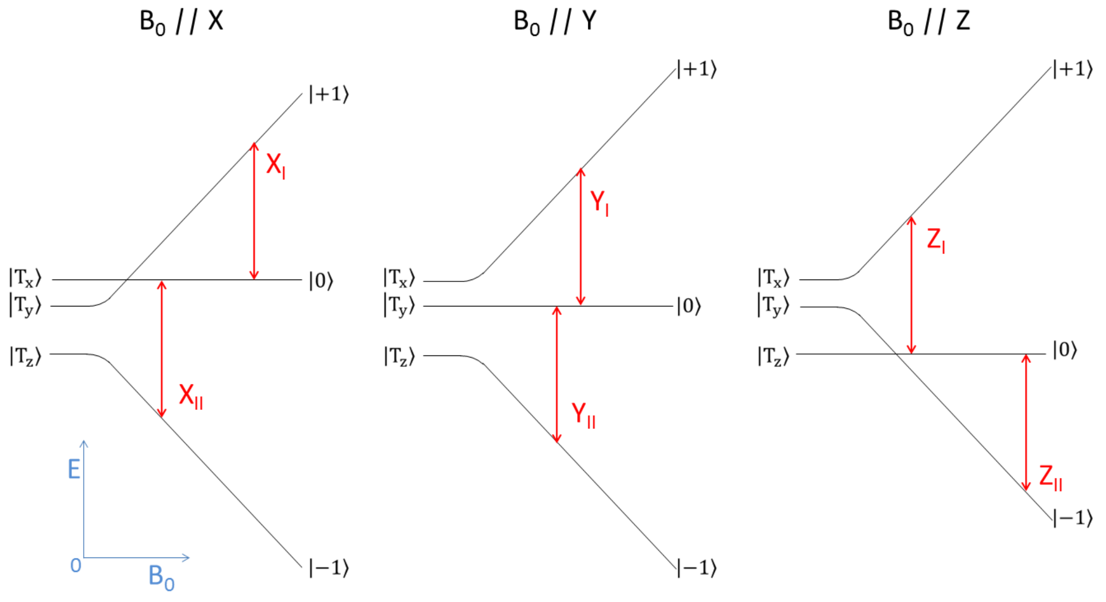

Figure S1. Scheme of electron spin energy levels of a chlorin triplet state in a magnetic field, with D>0 and E<0, for the canonical orientations as function of the magnetic field.

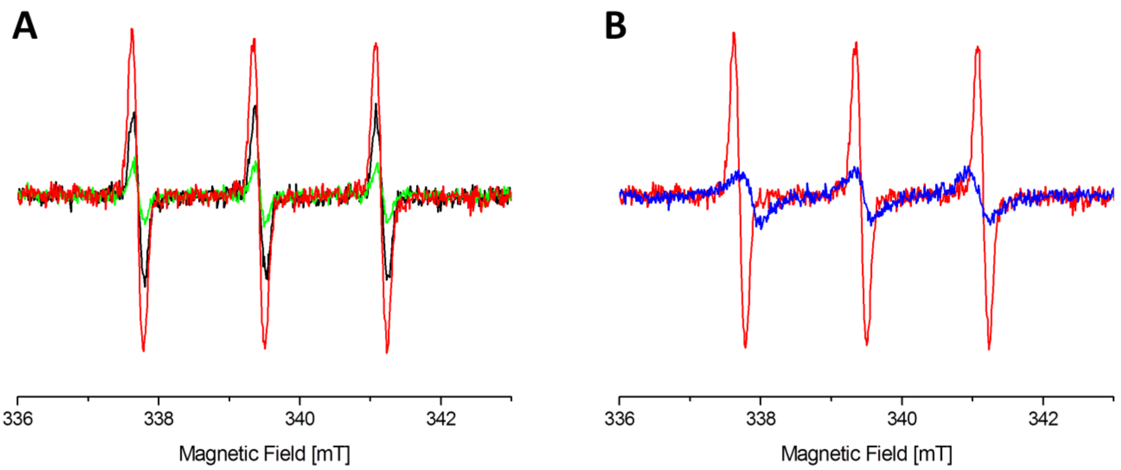

Figure S2. Spin-trap EPR measurements of singlet oxygen via TEMPO-formation after illumination at 150 µmol of photons m^{-2} s^{-1} for 60 min. Background signals were detected prior to illumination and subtracted. *(A)* Spin-trap EPR measurements on Chl *a* reconstituted WSCP complexes in 20 mM sodium phosphate pH 7.8 (black) and in the same buffer with the addition of 0.1% (w/v) DM (green), in comparison to Chl *a* in 0.1% (w/v) DM (red). *(B)* Comparison of spin-trap EPR measurements on Chl *a* (1 µg/mL) in 0.1% (w/v) DM (red) and in ethanol (blue).

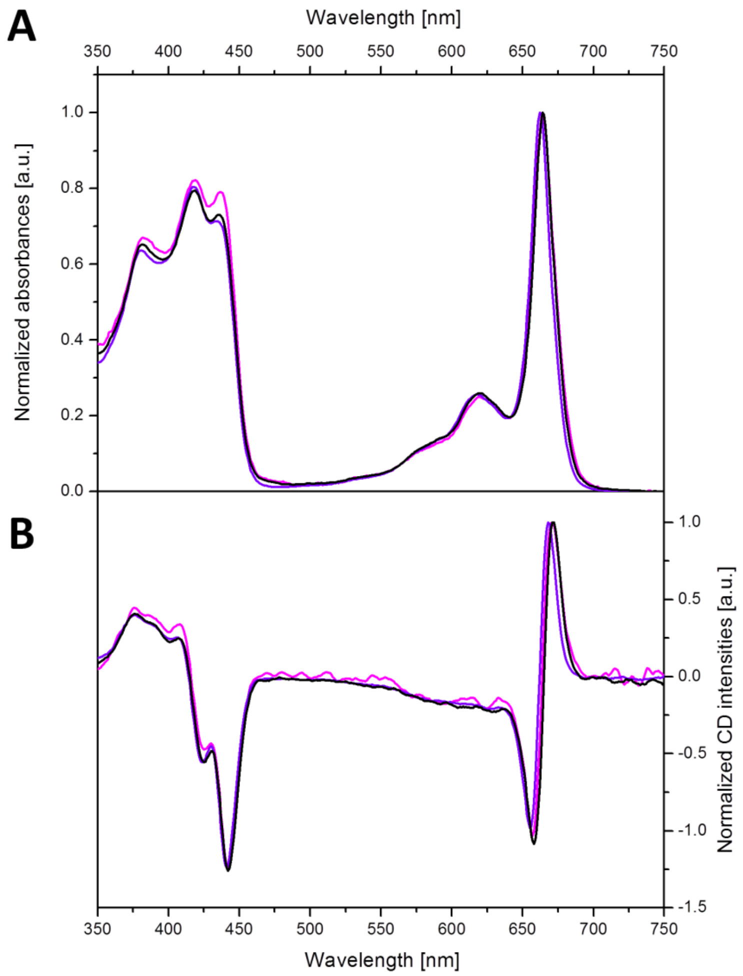

Figure S3. Absorption *(A)* and Vis-CD *(B)* spectra of Chl *a*-reconstituted WSCP (WT in black, W90F in magenta and W154F in violet). The spectra were normalized to their Q_y absorption maxima.

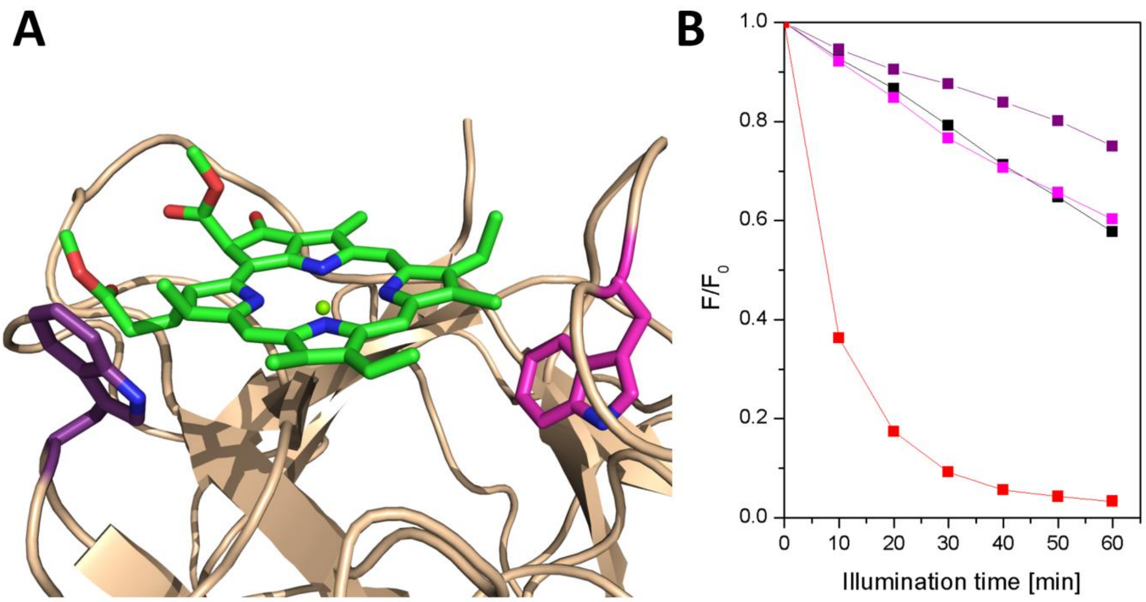

Figure S4. *(A)* Structure of *L. virginicum* WSCP (PDB entry 2DRE)[11] with the Chl *a* in green (phytyl omitted for clarity) and the apoprotein secondary structure in beige. Only a detail of the surrounding (within 10 Å) of the bound Chl is shown. Tryptophans W90 (magenta) and W154 (violet), in van der Waals contact with the Chl, are shown as sticks. *(B)* Photostability of Chl *a* reconstituted WSCP (WT, black; W90F, magenta; W154F, violet) in comparison to Chl *a* in 2 % (w/v) OG (red). The samples were illuminated with 500 µmol of photons m^{-2} s^{-1} for 0-60 min. Emission detected at the maximum of the emission spectrum, excitation at 410 nm. Ratio (F/F_0) of emission after (F) and before (F_0) illumination is plotted against illumination time.

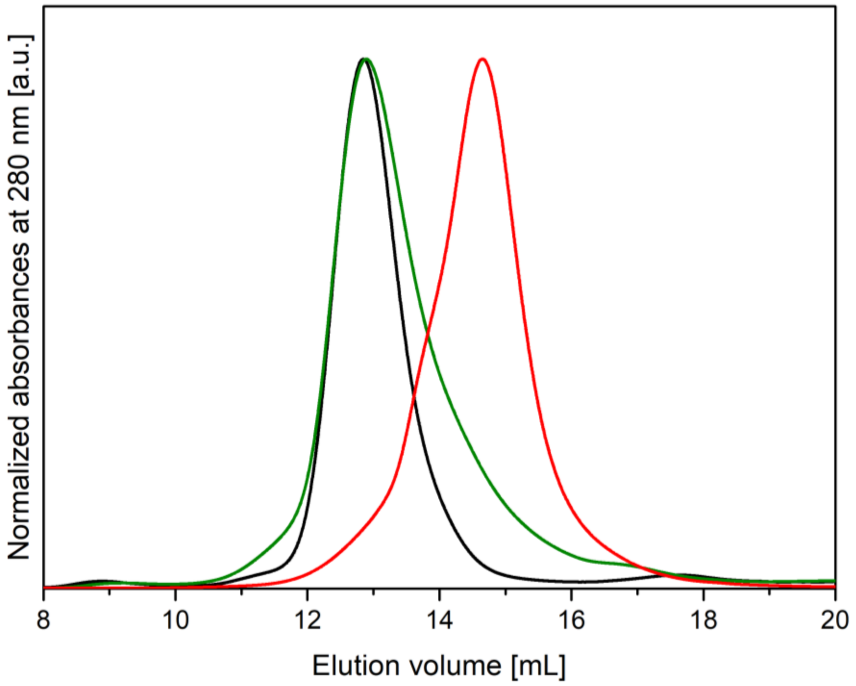

Figure S5. Size-exclusion chromatograms of apo-WSCP (red) and WSCP reconstituted with either Chl *a* (black) or Chlide *a* (dark green).

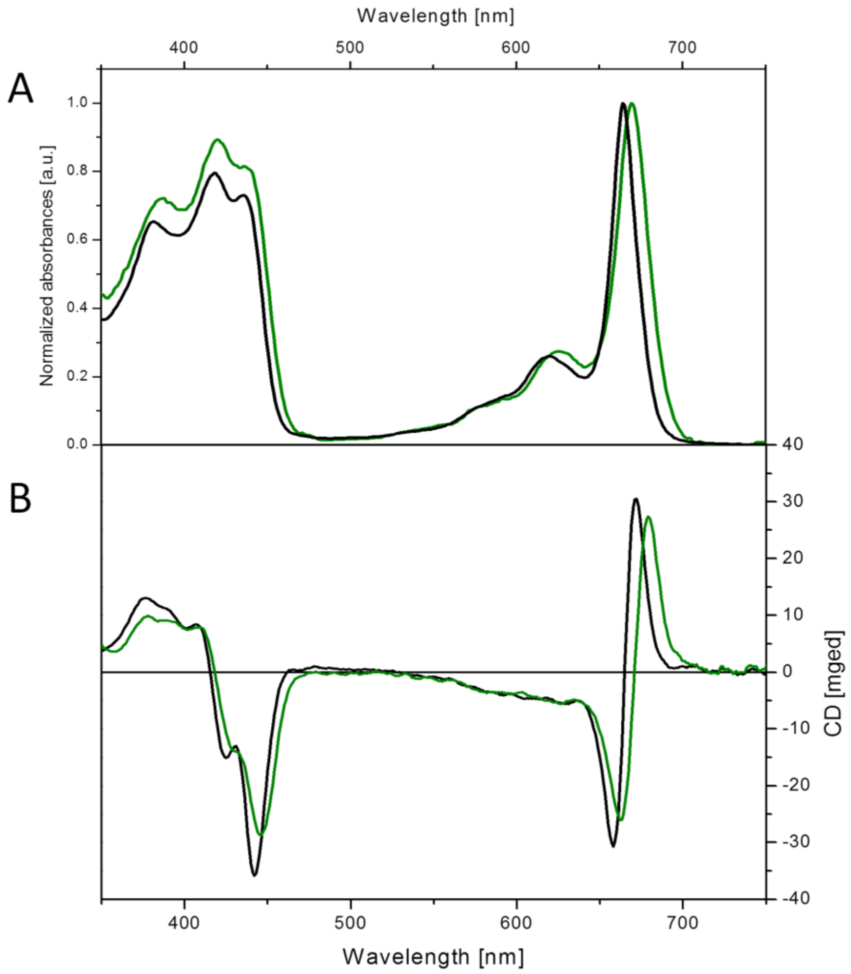

Figure S6. *(A)* Absorption spectra of WSCP reconstituted with either Chl *a* (black) or Chlide *a* (green). The spectra were normalized to their Q_y maxima. *(B)* Vis-CD spectra of WSCP reconstituted with either Chl *a* (black) or Chlide *a* (green). The spectra were normalized to the Q_y absorption band.

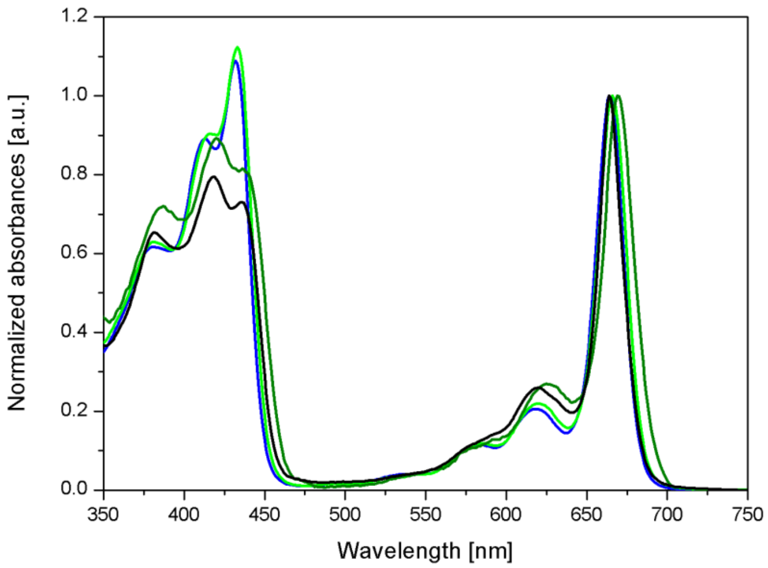

Figure S7. Comparison of absorption spectra of Chl *a* (blue) and Chlide *a* (light green) in 80 % (v/v) acetone with the spectra, reported also in figure 7 of the main text, of WSCP reconstituted with either Chl *a* (black) or Chlide *a* (dark green). The spectra were normalized to the Q_y maxima.

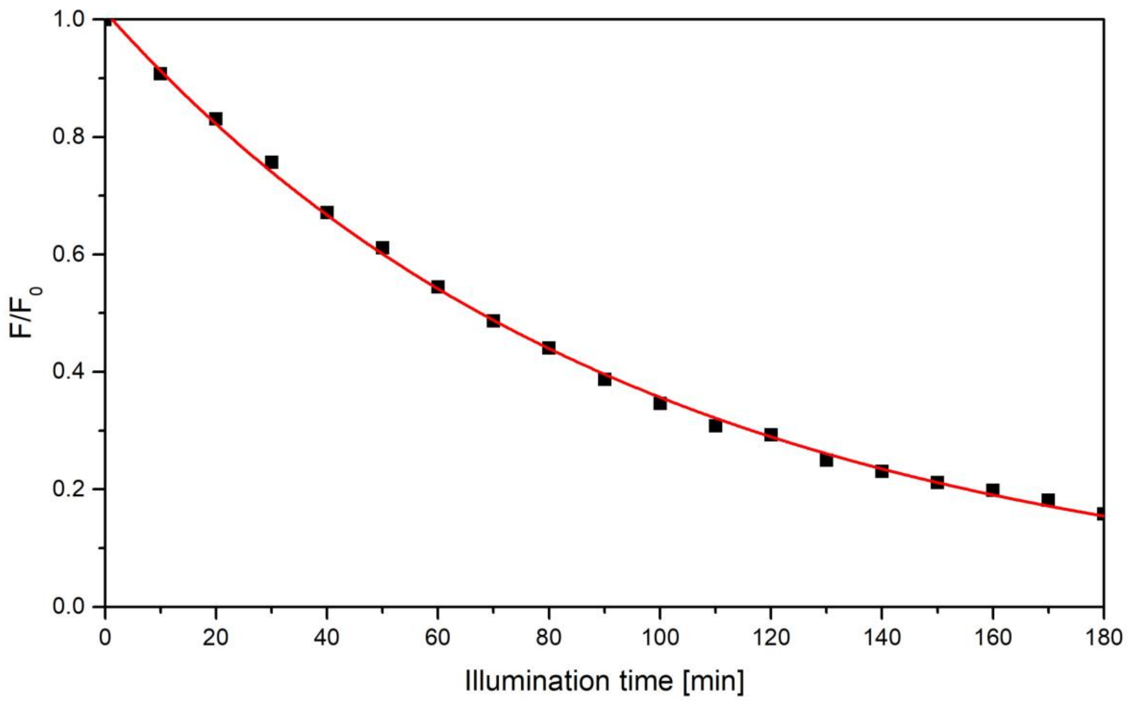

Figure S8. Photostability of Chl *a* reconstituted WSCP for long illumination times. The sample was illuminated with 500 µmol of photons m^{-2} s^{-1} for 0-60 min. Emission detected at the maximum of the emission spectrum, excitation at 410 nm. Ratio (F/F_0) of emission after (F) and before (F_0) illumination is plotted against illumination time. The mono-exponential fit is shown in red (t_c = 96 min).

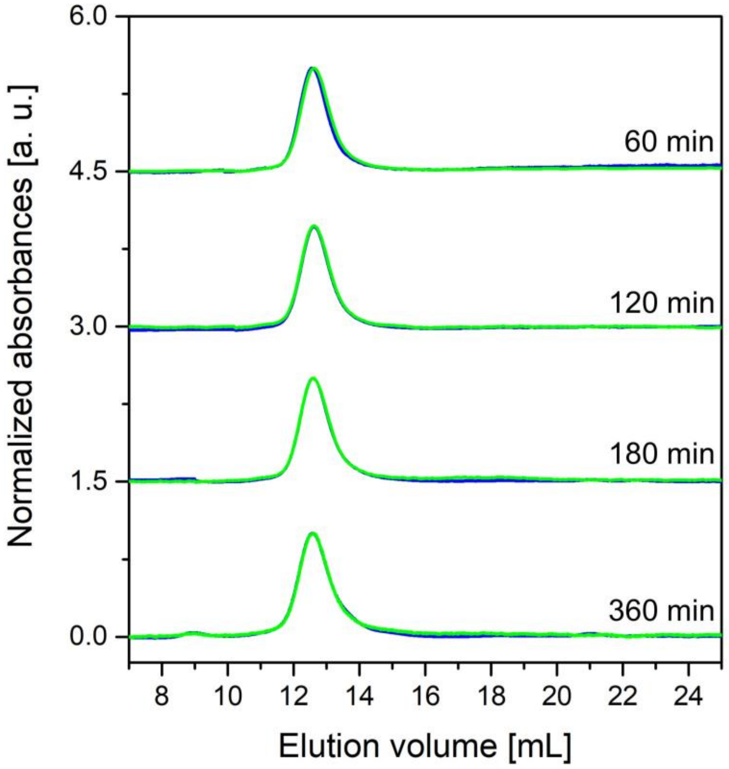

Figure S9. Assessment of Chl *a* reconstituted WSCP complex stability towards long illumination times. The sample was illuminated with 500 µmol of photons m^{-2} s^{-1} up to 6 hours. After the indicated time intervals (60 min, 120 min, 180 min and 360 min) complex integrity was validated by size-exclusion chromatography. Protein absorption was monitored at 280 nm (blue lines), Chl *a* absorption at 664 nm (green lines). The chromatograms have been vertically shifted for a better comparison.

Table S1. Parameters of the mono-exponential fits of the photobleaching curves reported in figure 5A. Parameters relative to the function $y = y_0 + A \cdot e^{-\frac{x}{t_c}}$.

	y_0	A	t_c
WSCP Chl *a*	0.2	0.8	96 min
WSCP Chlide *a*	0.1	0.9	32 min
Chl *a*	0.0	1.0	11 min
